# Association of Arterial Stiffness Index and Brain Structure in the UK Biobank: A 10-Year Retrospective Analysis

**DOI:** 10.14336/AD.2023.0419

**Published:** 2024-08-01

**Authors:** Elric Y Allison, Baraa K Al-Khazraji

**Affiliations:** Department of Kinesiology, Faculty of Science, McMaster University, Hamilton, ON, Canada

**Keywords:** Arterial stiffness, brain structure, brain function, cohort, cardiovascular health

## Abstract

Arterial stiffening and changes in brain structure both occur with normal aging and can be exacerbated via acquired health conditions. While cross-sectional associations exist, the longitudinal relationship between arterial stiffness and brain structure remains unclear. In this study, we investigated 1) associations between baseline arterial stiffness index (ASI) and brain structure (global and regional grey matter volumes (GMV), white matter hyperintensities (WMH)) 10-years post-baseline (10.4±0.8 years) and 2) associations between the 10-year change in ASI from baseline and brain structure 10-years post-baseline in 650 healthy middle- to older-aged adults (53.4±7.5 years) from the UK Biobank. We observed significant associations between baseline ASI and GMV (*p*<0.001) and WMH (*p*=0.0036) 10-years post-baseline. No significant associations between 10-year change in ASI and brain structure (global GMV *p=*0.24; WMH volume *p*=0.87) were observed. There were significant associations of baseline ASI in 2 of 60 regional brain volumes analyzed (right posterior superior temporal gyrus *p*=0.001; left superior lateral occipital cortex *p*<0.001). Strong associations with baseline ASI, but not changes in ASI over 10-years, suggest arterial stiffness at the entry point of older adulthood is more impactful on brain structure 10-years later compared to age-related stiffening. Based on these associations, we suggest clinical monitoring and potential intervention for reducing arterial stiffness should occur in midlife to reduce vascular contributions to structural changes in the brain, supporting a healthy trajectory of brain aging. Our findings also support use of ASI as a surrogate for gold standard measures in showing overall relationships between arterial stiffness and brain structure.

## INTRODUCTION

Changes in brain structure (e.g., grey matter atrophy, white matter lesions) accompany normal aging [1-10]. Accumulation of vascular risk factors may accelerate structural changes in the brain beyond what is expected with normal aging [11, 12]. Perturbations in vascular system integrity are associated with reduced cognitive function [13-15] and progression of neurodegenerative conditions such as vascular dementia and Alzheimer’s disease [16]. One proposed mechanism underlying vascular-related associations with brain structure is increased arterial stiffening [13, 15, 17], which can occur alongside health conditions (e.g., hypertension) or simply as part of the normal aging process [18-22]. In otherwise healthy individuals, the rate of arterial stiffening increases significantly after the age of 50 (2.05±0.03 m/s increase in pulse wave velocity (PWV)/decade; where PWV is a gold standard measure for arterial stiffening)[23]. Increased pulsatility and high-pressure blood flow secondary to vessel wall stiffening may lead to cerebral microvascular damage, contributing to the onset and progression of cerebrovascular damage and subsequent chronic reductions in cerebral blood flow (CBF) seen with aging and disease [24, 25].

Reductions in CBF are associated with grey matter (GM) atrophy and greater white matter hyperintensity (WMH) burden [11, 26]. The clinical relevance of GM atrophy and elevated WMH volume includes impaired neurological function and progression of neurodegenerative disorders [27-29]. Cross-sectionally, higher arterial stiffness is associated with reductions in global GM volume (GMV), elevated WMH volume, and cognitive deficits [12, 30, 31], although the longitudinal relationship between changes in arterial stiffness and changes in brain structure remains controversial [12, 32, 33]. Recent findings from the SMART-MR and Whitehall II studies observed no relationship between the rate of arterial stiffening and global brain volumes at the end point of the studies over 4 and 4.1±0.2 years, respectively [12, 32]. However, significant associations between arterial stiffening and reductions in regional CBF have been identified [32, 34]. Two possible explanations for a lack of relationship between changes in arterial stiffness and global brain structure are 1) the duration of 4 years may not capture brain structural adaptations to arterial stiffness changes and arterial stiffness changes may require longer duration of observation [12, 32], and 2) arterial stiffness-related reductions in CBF may result in a heterogenous pattern of GM atrophy [35]. A study conducted by Bown and colleagues identified significant effects of baseline arterial stiffness on occipital and hippocampal GMV, and temporal region WMH volume in a ~5 year follow up, but no associations with global brain structure [33]. A review from Fjell et al., suggests that the most drastic age-related reductions in GM occur in the frontal and temporal lobes, independent of any evidence of pathology [36]. Regional GM atrophy patterns associated with arterial stiffening remain unclear, but are important to consider as regional cortical atrophy is linked with domain-specific changes in brain function [28, 32, 37]. Furthermore, changes in brain structure associated with arterial stiffening may provide insight on one way in which vascular-related factors (i.e., arterial stiffening) contribute to cognitive deficits and progression of neurodegenerative diseases [13-16].

Via data from the UK Biobank, we aimed to investigate the temporal relationship between arterial stiffness and brain structure outcomes via two primary objectives in a single cohort of otherwise healthy middle and older aged adults. Our first objective aimed to extend on previous work by investigating the temporal relationship arterial stiffness and brain structure outcomes over a longer time span (4 years in Jochemson *et al.*; 4.1±0.2 years in Suri *et al.* ; 4.9 ± 1.6 years in Bown *et al. vs.* 10.4 ± 0.8 years in our study) in a group of healthy middle- and older-aged adults [12, 32, 33]. We examined the effects of baseline arterial stiffness, and the change in arterial stiffness over ~10 years on global brain structure. We hypothesized that both greater arterial stiffness at baseline, and greater changes in arterial stiffness would be associated with lower global GMV and greater WMH at 10 years post-baseline. Our second objective aimed to assess the temporal relationship between arterial stiffness and regional brain structure (regional cortical and subcortical GM volumes in 60 regions: 30 per hemisphere) 10 years post-baseline.

## MATERIALS AND METHODS

### Study Population

We analyzed data from the first full release of the UK Biobank database (released in 2012). The UK Biobank is a prospective study of over 500,000 participants recruited in 2006-2010 [38]. Data collected from the participants included questionnaires, physical measures, MRI imaging data, and ongoing hospital records. UK Biobank received ethical approval from the Northwest Multi-Centre Research Ethics Committee (REC reference: 11/NW/0382). All participants in the UK Biobank gave informed consent for the study via a touch-screen interface that required agreement for all individual statements on the consent form as well as the participant's signature on an electronic pad. In this process, all participants gave informed consent for data linkage as one statement requested consent for access to medical and other health-related records, the long-term storage and use of this and other information about the participants, also after incapacity or death, for health-related research. The UK Biobank consent form is available at: www.ukbiobank.ac.uk/media/05ldg1ez/consent-form-uk-biobank.pdf. UK Biobank has approval from the institutional review boards, namely, the North West Multi-centre Research Ethics Committee for the UK, from the National Information Governance Board for Health & Social Care for England and Wales, and from the Community Health Index Advisory Group for Scotland (www.ukbiobank.ac.uk/media/0xsbmfmw/egf.pdf). We acquired approval from the UK Biobank to access data based on the proposed research questions (application # 71652) as well as ethics approval from McMaster Research Ethics board (MREB#: 5833)(Hamilton, ON, Canada).

#### Study Design

To investigate the role of changes in arterial stiffness on brain structure, we included male and female middle- and older-aged adults (53.4 ± 7.5 years; age range: 40-70 years) at two time points (1-baseline and 2-imaging visit 2) separated by 10.4 ± 0.8 years (range 8.8-12.2 years). Participants were excluded from the present analysis if they reported having been diagnosed with diabetes mellitus, hypertension, cardiovascular disease, stroke, angina, neurodegenerative diseases (dementia, Parkinson’s disease, Alzheimer’s disease), brain cancer, brain haemorrhage, brain abscess, aneurysm, cerebral palsy, encephalitis, head injury, nervous system infection, multiple sclerosis, head, or neurological injury. Only participants that met all the eligibility criteria, as well as having data for ASI at baseline and second imaging visit (i.e., ~10 years following baseline), were included in study analyses. Participants were also excluded from analyses if they were missing any data for primary measures including both baseline ASI and ASI at the 10-year end point, or if they were missing data for any of our primary outcome measures of 60 regional GMVs (50 cortical, 10 subcortical), global GMV, and WMH volume at the 10-year endpoint. Following all filtering of subjects to match inclusion criteria, we excluded >500,000 subjects from the full UK Biobank cohort and included N=650 participants in our final analyses. To investigate the role of arterial stiffness on changes in brain structure and function, we used the change in ASI over approximately a decade as the independent variables in Objective 1 and 2 and accounted for individual differences in time between visits as a covariate.

### Measures

#### Pulse wave arterial stiffness index (ASI)

ASI was measured during the first visit to the assessment center using the PulseTrace PCA2 (CareFusion, San Diego, CA) (Field-ID 21021) in 169,829 participants from 2006 until 2010 as part of the UK Biobank cohort study. The PulseTrace PCA2 uses finger photoplethysmography to obtain the pulse waveform during a 10- to 15-s measurement using an infrared sensor clipped to the end of the index finger. The measurement was repeated on a larger finger or on the thumb if less than two thirds of the waveform was visible on the display of the PulseTrace PCA2 device, or if the waveform did not stabilize within 1 minute after clipping the infrared sensor to the end of the index finger (protocol: https://biobank.ndph.ox.ac.uk/showcase/showcase/docs/Pulsewave.pdf). The shape of the waveform is directly related to the time it takes for the pulse wave to travel through the arterial tree in the lower body, and to be reflected to the finger. ASI, while not the gold standard measurement technique for the assessment of arterial stiffness, has been demonstrated to be a simple, quick, and easily reproducible alternative approach for the measurement of arterial stiffness that is favourable for large scale population-based studies such as the UK Biobank. The strength of relationship between carotid-femoral PWV (applanation tonometry) and ASI is higher than other measures of arterial stiffness such as PWV and arterial compliance (r=0.65 vs. r=0.47) [39, 40]. Correlation between ASI and PWV was not possible in the UK Biobank data, as there was no overlap in participants who had *both* measures of arterial stiffness assessed.

#### Magnetic resonance imaging (MRI) data and image processing

In 2014, UK Biobank began inviting back 100,000 of the original volunteers for brain, heart, and body imaging. Imaging data for 10,000 volunteers has already been processed and made available for further research [41]. The included MRI sequences are T1 (Section T1 pipeline), T2-FLAIR (Section T2 FLAIR pipeline) anatomical scans. All brain MRI data were acquired on the same 3T Siemens Skyra scanner, following a freely available protocol (www.fmrib.ox.ac.uk/ukbiobank/protocol/V4_23092014.pdf). In 2014, UK Biobank began inviting back 100,000 of the original volunteers for brain, heart, and body imaging. Imaging data for 10,000 volunteers has already been processed and made available for further research [41]. Data were acquired with a standard Siemens 32-channel head coil. 3D T1-weighted (in plane acceleration factor = 2, voxel size = 1x1x1mm, field of view = 208x256x256, TI = 880ms, TR = 2000ms, acquisition time = 4:54 minutes) and 3D T2-weighted FLAIR volumes (in plane acceleration factor = 2, voxel size 1.05x1x1mm, field of view = 192x256x256, TI = 1800ms, TR = 5000ms, acquisition time = 5:52 minutes) were acquired in sagittal orientation. Brain imaging data were processed by the UK Biobank team and made available to approved researchers as imaging-derived phenotypes; the full details of the image processing and quality control pipeline are available in documentation of the brain MRI image processing methods used in the UK Biobank (https://biobank.ndph.ox.ac.uk/ukb/ukb/docs/brain_mri.pdf) and publication [41]. In brief, using tools from the FSL MRI analysis library (https://fsl.fmrib.ox.ac.uk/fsl/fslwiki), imaging-derived phenotypes for GM and WHM volumes were extracted using the FMRIB Automated Segmentation Tool (FAST) (https://fsl.fmrib.ox.ac.uk/fsl/fslwiki/FAST) and the Brain Intensity Abnormality Classification Algorithm (BIANCA)(https://fsl.fmrib.ox.ac.uk/fsl/fslwiki/BIANCA), respectively. Imaging-derived phenotypes used in analyses included global GMV and WMH volume, and 60 regional GMV Imaging-derived phenotypes (50 cortical, 10 subcortical) derived using parcellations from the Harvard-Oxford cortical and subcortical atlases and Diedrichsen cerebellar atlas.

#### Statistical Analysis

For Objectives 1 and 2, the temporal relationship between ASI and brain structure was examined using robust GLMs. All statistical analyses were conducted in the freely available statistical software R (https://cran.r-project.org), and we used the *robust* package (https://cran.r-project.org/web/packages/robust/robust.pdf) to implement the robust GLMs. For Objective 1, baseline ASI and DASI over 10.4 ± 0.8 years were treated as the independent variables in two separate analyses, with both global GMV and WMH volume at the 10-year end point as the outcome variables. For Objective 2, baseline ASI was treated as the independent variable with GM volumes in 50 cortical and 10 subcortical brain regions as outcomes of interest. Covariates for both Objectives 1 and 2 included age, sex, and time between visits in years (Equation 1 and Equation 2, respectively).

Where appropriate, significant p-values were adjusted using the Holm-Bonferroni post-hoc test for multiple comparisons. Significant findings were reported when the adjusted p-value was below the set alpha threshold of (*p* = 0.05/61 for changes in global and regional GM volumes). Raw and *post-hoc* corrected *p* values for cortical and subcortical GM volumes can be found in Supplemental Materials 1. All values are reported as mean ± SD unless otherwise stated.

Equation 1 $$\text{Outcome}\sim {\text{Baseline}}_{\text{ASI}}+\text{Convariates}+\text{error}$$

Equation 2 Outcome~ΔASI+Convariates+error

**Table 1:** **Demographics of individuals included in analyses (mean±SD unless otherwise stated)**. Body Mass Index (BMI); Blood pressure (BP); Self-reported physical activity (PA); Hormone replacement therapy (HRT); Glycated Hemoglobin (HbA1c).

*N=650*	*M*	*SD*
Female (n)	345	-
Male (n)	305	-
Age at baseline (years)	53.4	7.5
Time between assessments (years)	10.4	0.8
Height (cm)	170.7	9.4
Weight (kg)	74.1	14.0
BMI	26.2	3.9
Body Fat (%)	29.5	8.1
Waist-to-hip Ratio	0.85	0.1
Arterial Stiffness Index at baseline (m/s)	8.8	2.8
Change in Arterial Stiffness Index (m/s)	0.18	3.9
Systolic BP (mmHg)	133.4	17.7
Diastolic BP (mmHg)	79.9	10.1
Mean Arterial Pressure (mmHg)	97.7	11.7
Minutes walking per week (min/week)	1105.8	1149.2
Moderate intensity exercise per week (min/week)	907.4	1154.0
Vigorous intensity exercise per week (min/week)	774.4	1088.5
Medications (N / %)		
Cholesterol Lowering Medication	13	2
Anti-hypertensives	2	0.3
HRT	23	3.5
Oral Contraceptives	7	1.1
HbA1c (mmol/mol)	34.5	3.8
Global Grey Matter Volume (mL)	794.1	46.7
White Matter Hyperintensities (mL)	4.5	5.3

## RESULTS

Demographics and outcome measures can be found in Table 1. We included 650 otherwise healthy male and female middle- and older-aged adults in our final working sample. Females accounted for 53% participants included in analyses. The age of participants at baseline was 53.4 ± 7.5 years, and the time interval between baseline and the second imaging visit was 10.4 ± 0.8 years. The mean systolic and diastolic blood pressure values in the sample at baseline was 133.4 ± 17.7mmHg and 79.9 ± 10.1mmHg, respectively. ASI at baseline was 8.8 ± 2.8m/s, and the change in ASI over ~10 years was 0.18 ± 3.9m/s. Of 650 included subjects, at time of recruitment only 13 (2%) reported taking cholesterol lowering medications, 2 (0.3%) reported taking anti-hypertensives, 23 (3.5%) reported using hormone replacement therapy, and 7 (1.1%) reported taking oral contraceptives.

**Figure 1.**
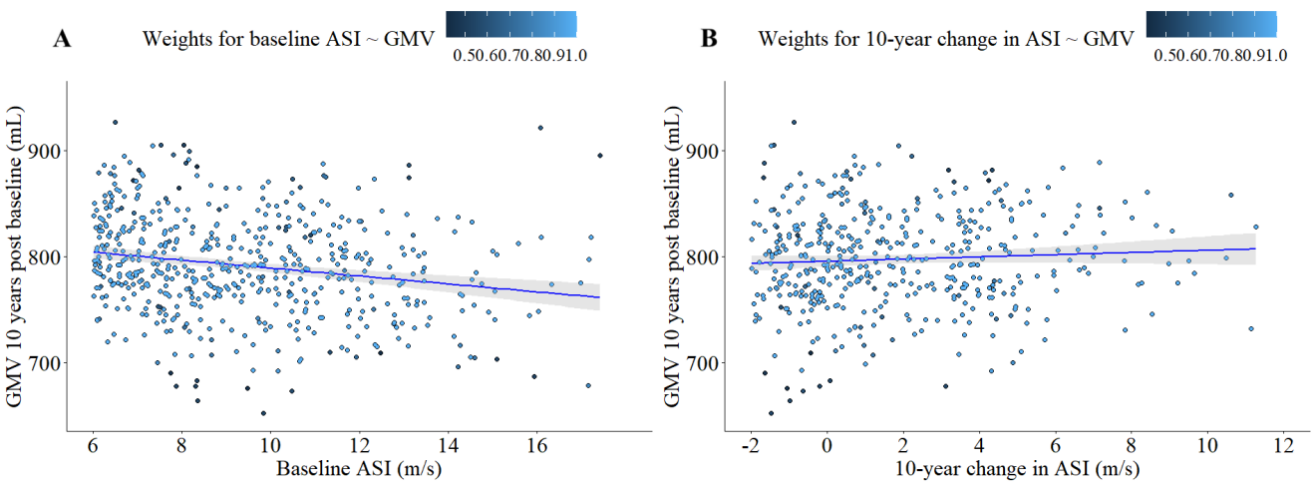
**Longitudinal relationship between ASI and global GMV**. (**A**) Association of baseline ASI (m/s) and global GMV 10.4 ± 0.8 years post-baseline (N = 650). (**B**) Association of DASI and global GMV 10.4 ± 0.8 years post-baseline (N = 650). Regression line and 95% confidence interval for both figures 1A and 1B is visualizing unadjusted Robust Generalized Linear Regression for visualization purposes. Weights applied to each data point are derived from adjusted Robust Generalized Linear Models used in statistical analyses (Table 2).

### Objective 1. Association between ASI and Global Brain Volumes.

*Association between ASI and Global GMV.* Our analyses found a significant association (*p* < 0.001) between baseline ASI on global GMV at the 10-year endpoint after correcting for age, sex, and time between baseline and final visits (Fig. 1A). Sex, age at baseline, and time between baseline and the final visit were significantly associated with global GMV (Table 2). Robust GLM models for baseline ASI and global GMV weighed 540 of the 650 subjects = 1, and the remaining 110 residuals were reweighed according to their proximity to model fit. Weights for the remaining 110 subjects ranged between 0.42 and 0.99. Compared to baseline ASI, we found no association between DASI and global GMV 10-years post-baseline (*p* = 0.24; Fig. 1B), whereas the effects of sex, age, and time between visits remained significant in the model assessing effects of DASI on global GMV. In the DASI model 536 of 650 robust GLM fits were given weights = 1, while the remaining 114 residuals were assigned weights between 0.41 and 0.99.

**Table 2:** **Robust GLM statistical summary of (A) Association between baseline Arterial stiffness index and (B) 10-year change in ASI on global grey matter volume (GMV) in a sample of otherwise healthy middle- and older-aged adults, corrected for age, sex, and time between visits (years) (N = 650)**. Significant associations are highlighted in bold.

A	Global GMV (mL)			
	B	95% CI	Uncorrected *p*	Corrected *p*
Baseline ASI (m/s)	-0.26	-0.29; -0.23	**<0.001**	**<0.001**
Age (years)	-3.76	-3.90; -3.88	**<0.001**	**<0.001**
Sex	-19.91	-20.07; -19.74	**<0.001**	**<0.001**
Time between visits (years)	-2.49	-5.59; -5.39	**<0.001**	**<0.001**
**B**				
DASI (m/s)	0.012	0.0099; 0.014	0.24	-
Age (years)	-3.92	-4.03; -3.81	**<0.001**	**<0.001**
Sex	-20.12	-20.14; -20.10	**<0.001**	**<0.001**
Time between visits (years)	-5.52	-5.62; -5.42	**<0.001**	**<0.001**

**Figure 2.**
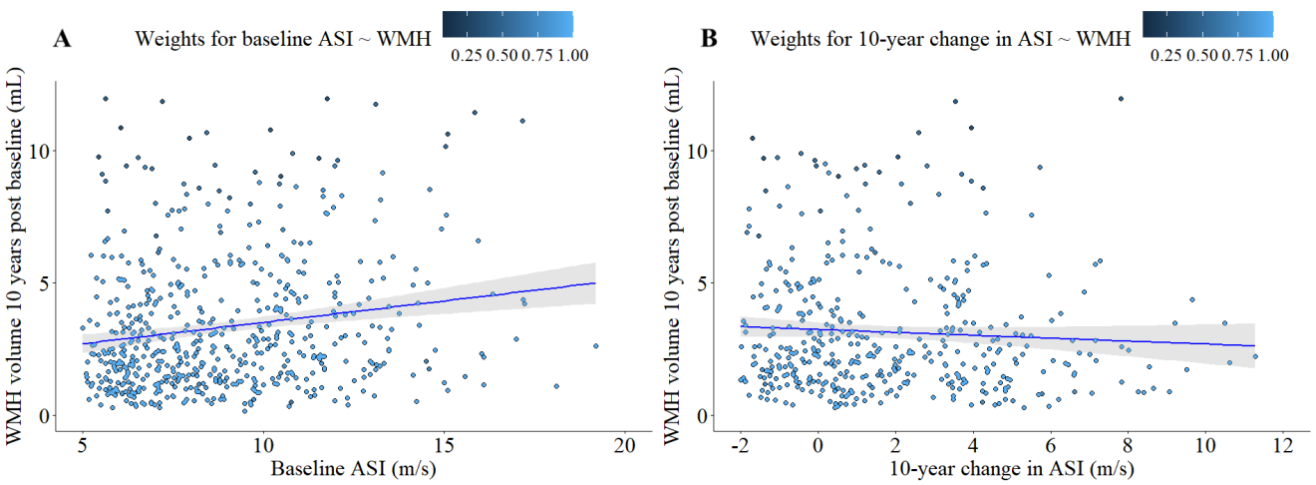
**Longitudinal relationship between ASI and global WMH**. (**A**) Association of baseline ASI (m/s) and global WMH 10.4 ± 0.8 years post-baseline (N = 650). (**B**) Association of ∆ASI and global WMH 10.4 ± 0.8 years post-baseline (N = 650). Regression line and 95% confidence interval for both figures 2A and 2B is visualizing unadjusted Robust Generalized Linear Regression for visualization purposes. Weights applied to each data point are derived from adjusted Robust Generalized Linear Models used in statistical analyses (Table 3).

*Association of ASI and WMH volume.* We identified a significant effect of baseline ASI on WMH volume at the 10-year end point after correcting for covariates of sex, age, and time between visits (*p* = 0.0036) (Fig. 2A). Robust GLM models for baseline ASI and WMH volume weighed 525 of the 650 subjects = 1, and the remaining 125 residuals were reweighed between 0.08 and 0.99. Our analyses found no effect of DASI on WMH volume (*p* = 0.871) (Table 3) after correcting for sex, age, and time between visits (Fig. 2B). 523 subjects were assigned a weight = 1, while the remaining 127 were assigned weights ranging from 0.08 to 0.99 in the DASI model.

Based on our findings from *Objective 1*, where no associations between changes in ASI and global brain structure were observed, only the effects of baseline arterial stiffness on regional brain volumes are reported in *Objective 2*.

**Table 3.** Robust GLM statistical summary of (A) Association between baseline Arterial stiffness index and (B) 10-year change in ASI on whole brain white matter hyperintensity volume (WMH) in a sample of otherwise healthy middle- and older-aged adults (N = 650). Significant associations are highlighted in bold.

A	WMH volume (mL)			
	B	95% CI	Uncorrected *p*	Corrected *p*
Baseline ASI (m/s)	0.05	0.02; 0.08	0.0036	-
Age (years)	0.16	0.15; 0.17	**<0.001**	-
Sex	0.54	0.38; 0.70	**<0.001**	-
Time between visits (years)	-0.05	-0.15; 0.05	0.35	-
**B**				
DASI (m/s)	-0.002	-0.02; 0.02	0.87	-
Age (years)	0.16	0.15; 0.17	**<0.001**	-
Sex	0.59	0.44; 0.75	**<0.001**	-
Time between visits (years)	-0.05	-0.15; 0.05	0.30	-

### Objective 2. Effects of ASI on Regional Brain Volumes.

Prior to *post-hoc* Holm-Bonferroni corrections for family wise comparisons, there were significant effects of baseline ASI on 7 GM regions (5 cortical, 2 subcortical) after adjusting for age, sex, and time between visits. These regions included the left hemisphere frontal pole (uncorrected *p =* 0.032), right hemisphere superior frontal gyrus (uncorrected *p* = 0.0422), right hemisphere middle frontal gyrus (uncorrected *p* = 0.039), right hemisphere posterior superior temporal gyrus (uncorrected *p* < 0.001), left hemisphere superior lateral occipital cortex (uncorrected *p*<0.001) left hemisphere anterior parahippocampal gyrus (uncorrected *p* = 0.012), and the right hemisphere parahippocampus (uncorrected *p* = 0.026). After *post-hoc* correction, only the right hemisphere posterior superior temporal gyrus (corrected *p* = 0.001) and the left hemisphere superior lateral occipital cortex (corrected *p* < 0.001) remained significant. Robust GLM applied weights to residuals ranging from 0.7 to 0.99. Statistical summary (raw and corrected *p* values) for each cortical and subcortical region analyzed can be found in Supplemental Table 1. All robust GLM statistical outputs are available upon request.

## DISCUSSION

Arterial stiffness begins to increase after midlife and worsens in later life [42]. Increased arterial stiffness reduces the ability to dampen pulse pressure, which enables transmittance of high pressure and pulsatile stress to distal microvasculature [43]. The brain is a highly vascularized organ, supplied by a low-resistance arterial network [44], with tight coupling between blood flow and metabolic demand given the limited fuel reserves [45]. With an increase in arterial stiffness, the brain becomes susceptible to microvascular damage [46, 47], vascular endothelial dysfunction [48], compromised blood brain barrier which can impact neurodegeneration [49], microbleeds and white matter lesions [50, 51]. Heightened arterial stiffness can also impact cerebral blood flow regulation [52] leading to insufficient matching of blood flow to neuronal metabolic demand and subsequent brain atrophy [53, 54].

In this study, we aimed to build on previous work investigating the temporal relationship between arterial stiffness and global brain structure by increasing the time course of interest (~4 years in the SMART-MR and Whitehall II imaging sub-study, 5 years in the Vanderbilt Memory and Aging study) [12, 32, 33] vs. ~10 years in our study using the UK Biobank dataset) in a cohort of otherwise healthy adults (N = 650). To our knowledge, this is both the longest study duration and the largest cohort in a single study investigating the relationship between arterial stiffness and brain structure to date in a healthy population of adults. The findings for each objective are discussed herein.

### Objective 1. Association Between ASI and Global Brain Volumes.

Following robust GLM analyses, we observed significant effects of baseline arterial stiffness (measured by ASI) on global brain structure (global GMV, WMH volume) a decade post-baseline, while observing no effects of the ~10-year changes in arterial stiffness (DASI) on brain structure. The absence of association of DASI and global GMV or WMH volume suggest that healthy adults with lower levels of arterial stiffness at baseline maintain structural integrity of the brain over 10 years despite age-related increases in arterial stiffness.

The significant associations between arterial stiffness and regional CBF in the Whitehall II imaging sub-study, taken alongside cross-sectional associations between arterial stiffness and brain structure, support existing research highlighting that stiffness-related reductions in CBF precede changes in brain structure [12, 30-32, 55]. In subclinical populations where stiffness-related reductions in CBF can be modest, the associated changes in GMV or WMH may take years to manifest or detect with current imaging and analysis approaches. Thus, our interpretation is that arterial stiffness at the entry point into older adulthood, and potentially longer exposure to higher arterial stiffness, holds significantly more impact on brain health than changes in arterial stiffness that occur with aging.

Our findings as they pertain to baseline arterial stiffness (significant effects on global GMV and WMH volume) conflict with the findings from the SMART-MR and Whitehall II studies, where no relationship between b-stiffness or baseline pulse wave velocity (PWV), respectively, on brain volumes (GMV or WMH volume) 4 years later was identified [12,32]. Our results also conflict with the findings from the Vanderbilt Aging and Memory study, where baseline arterial stiffness was not significantly associated with global GMV or WMH 4.9 ± 1.6 years later [33]. While they found no significant relationships with global GMV, the Vanderbilt study did identify significant associations between baseline arterial stiffness and temporal region WMH volume [33]. Cross-sectional associations between arterial stiffness and changes in brain structure are well-established and have been frequently reported [12, 30, 31, 55]; however, to our knowledge, we provide novel information on the relationship between arterial stiffness and structural changes in the brain over a longer period of time (i.e., 10 years). Previous work assessing arterial stiffness impacts on brain outcomes over 4-5 years may not have captured changes in global brain structure that may present over a longer duration (~10 years in our study). As arterial stiffness is considered a subclinical measure of vascular health, and seemingly involves intermediate steps of cerebral microvascular damage and reductions in cerebral blood flow, its effects on the brain (e.g., GM atrophy or increased WMH) likely require long-term chronic exposure. This is supported by the lack of relationship between the 4- and 10-year change in stiffness and end-point brain structure in the Whitehall II imaging sub-study and the present study, respectively [32]. Indeed, these findings suggest that age-related changes to vascular structure and function likely do not have immediately detectable effects on brain structure.

We observed significant associations between baseline age and both global GMV and WMH volumes at the 10-year end point. Unsurprisingly, a higher age at baseline was associated with lower GMV and greater WMH volume at 10-year follow-up. We observed that each year increase in age at baseline was associated with a 0.5% reduction in global GMV at 10-year follow-up, which is comparable to the consensus range of reductions in global GMV/year in healthy older adults found in other studies (~0.2-0.7%/year) [2, 3, 12, 56, 57] Similarly, we observed that per year increase in age at baseline contributed to a 0.16mL increase in WMH volume at 10-year follow-up, within the frequently reported range of ~0-0.4mL increase/year expected with normal aging [5, 6, 9, 58-60]. In addition to associations with age, we also found significant associations between biological sex and brain structure. Men demonstrated significantly lower global GMV at the 10-year end point compared to women, aligning with several previous works [61-63]. We also identified a significant association of sex on WMH volume, where men had significantly higher WMH volume compared to women at the 10-year end point. These findings are inconsistent with evidence suggesting greater WMH volume with advancing age in post-menopausal women compared to men [64-67]. Possible explanations for the contrast between our findings and literature may be due to stringent exclusion criteria, resulting in our sample being an exclusively healthy group of middle- and older-aged adults. Another potential reason for inconsistent findings may be the heterogenous nature of the sample, particularly as it pertains to females. Specifically, the age range in the UK Biobank sub sample used for analyses of 40-70 years old likely includes both pre- and post-menopausal women. It is possible that pre-menopausal women, who still benefit from the vasoprotective effects of estrogen, may influence the overall trend in the present study. Future studies may conduct intentional analysis on how arterial stiffness impacts brain structural outcomes differently between pre- and post-menopausal women.

Finally, our observed associations between baseline ASI and brain structure align with cross-sectional evidence that primarily has measured arterial stiffness using the gold standard approach of PWV. Moderate agreement between ASI and PWV was shown in another study (r=0.65; N=87)[39]. Given the alignment of our brain outcome findings when using ASI as surrogate measure of arterial stiffness vs when using the gold standard PWV measure in other studies [30-33], we suggest ASI has similar capacity to PWV in showing overall trends of how arterial stiffness impacts brain structure, while having the advantage of easier data collection compared to PWV approaches.

### Objective 2. Association Between ASI and Regional Brain Volumes.

The present study aimed to investigate the relationship between arterial stiffness and changes in regional cortical and subcortical GMV over an average of ~10-years by building on previous studies that had observed these effects over 4-5 years [12, 33, 39]. Prior to *post-hoc* correction, we saw a significant association between baseline ASI and brain volume in 5 cortical and 2 subcortical brain regions. The cortical regions included two frontal regions (left hemisphere frontal pole, right hemisphere superior frontal gyrus), one temporal region, and one occipital region. This aligns with the literature, where significant associations between PWV and reductions in regional CBF in the frontal, temporal, parietal, and occipital regions have been observed [32, 34]. The subcortical regions associated with baseline ASI included the left hemisphere anterior parahippocampal gyrus and the right hemisphere parahippocampus. Only the right hemisphere posterior superior temporal gyrus and the left hemisphere superior lateral occipital cortex survived Holm-Bonferroni correction for family-wise comparisons. Our findings partially agree with the work from Bown *et al.,* where regions most significantly associated with baseline arterial stiffness in a 4.9 ± 1.6 year follow-up included occipital and hippocampal regions [33]. Reductions in GMV in parahippocampal regions is of clinical relevance, as GM atrophy in these regions has been associated with early stage Alzheimer’s disease [68, 69]. Despite observing significant associations between baseline ASI and global GMV, at the regional level there were no clear spatial atrophy patterns identified. This may be attributed to the small, albeit significant (b-0.26mL 95% CI -0.29 - -0.23mL) effect of baseline ASI on global GMV. The significant effects of baseline ASI on global brain structure are spread across 139 available imaging-derived phenotypes from the UK Biobank (60 included in the present analysis), resulting in small model estimates for each region. Thus, future studies may focus on clustering brain regions to examine the effects of arterial stiffness on functionally connected cortical and subcortical networks as opposed to discrete regions.

While few associations between ASI on regional brain structure were identified, we did observe significant relationships between age and regional brain volumes after correction in our sample. Cortical regions most strongly associated with age at baseline included 6 frontal regions, 5 temporal regions, 4 parietal regions, and 4 occipital regions. These findings agree with much of the literature related to age-related changes in brain structure, where frontal and temporal cortical regions are most vulnerable to age-related GM atrophy [1, 35, 70, 71]. These findings support the “last in, first out” hypothesis highlighted by Fjell et al, suggesting that more advanced and late maturing brain regions responsible for higher level cognitive tasks are the most vulnerable to age-related changes in structure [36]. We also found significant associations between age and GM volume in 7 subcortical regions. Our patterns of age-related associations in subcortical brain volumes are in agreement with several other studies that have shown subcortical vulnerability to advancing age [72-74].

We identified significant associations between sex and GMV in 16 cortical regions and 12 subcortical regions after correction. Sex differences at the cortical level included 6 frontal, 4 occipital, 3 temporal, and 3 parietal regions. Regional sex differences in cortical and subcortical GM volumes is consistent with the literature, and has been confirmed via meta-analyses, as well as other large-scale population-based studies [75, 76]. While sex differences were identified in our analyses, these findings are interpreted with caution as biological sex was included as a covariate, but no sex-specific analyses were conducted.

Our study is not without limitations. While ASI is a more feasible approach for large-scale population studies, and perhaps the clinical assessment and monitoring of vascular stiffness, our independent variable (arterial stiffness) was not measured using the gold standard approach of PWV. Using PWV for large-scale data collection (as in the UK Biobank) would help improve validity and reliability of the independent variable and its associations on brain structure, although our ASI data follows similar trends to previous work that used PWV [30-33]. The CBF data were not available to us in the UK Biobank, which would have supported physiological rationale for links between arterial stiffness and changes in brain structure. Despite not having CBF measures for this study, we rationalized our second objective based on previous findings showing associations of arterial stiffness and regional changes in CBF [32, 34]. Chronic reductions in CBF are associated with atrophy of neural tissues [24, 25]. Thus, relating arterial stiffness, CBF, and regional brain structure longitudinally (~10 years) in a large population would improve interpretation of findings. As previously mentioned, the use of cortical and subcortical networks as outcomes instead of isolated brain regions may more clearly elucidate regional effects of arterial stiffness on the brain. Inclusion of only healthy older adults also limits generalizability of our findings as chronic conditions, use of medication, and comorbidities are prevalent in older adults. However, our choice of participant pool was predicated on knowledge that arterial stiffening is impacted by several disorders [77, 78]. As such, understanding arterial stiffness impacts on brain structure in healthy aging required careful consideration of a relatively healthy group of adults. The effects of subclinical arterial stiffness on the brain in otherwise healthy middle- and older-aged adults is likely less than one might see in other specialized populations. Future studies should aim to include individuals across the health spectrum, providing a broader understanding of the relationship between vascular health and brain structure and function. While sex was considered by inclusion as a covariate, we did not run sex-specific analyses. Sociodemographic factors including socioeconomic status, ethnicity, and education, are established cardiovascular risk factors, and were not considered in the present study [79-81].

### Perspectives

We report several novel findings in this cohort of 650 community-dwelling healthy middle- and older-aged adults from the UK Biobank. We found that baseline arterial stiffness, not changes in arterial stiffness with aging, was associated with lower whole brain GMV and greater WMH volume. These findings suggest that those who have lower baseline arterial stiffness better maintain brain structure when compared to those who enter older adulthood with greater levels of arterial stiffness. As it relates to the association between arterial stiffness and regional brain structure, the brain regions most strongly associated with arterial stiffness were aligned with the reductions in regional CBF reported elsewhere, supporting the potential role of arterial stiffness-related cerebral hypoperfusion in brain structural changes [32]. Following *post-hoc* correction, we observed no clear spatial patterns of the associations between arterial stiffness and regional brain volumes. This may be attributed to the small effects of arterial stiffness on global brain structure spread over multiple regions, likely making the effects of arterial stiffness on discrete brain regions difficult to detect. Additionally, despite not using the consensus gold standard of PWV, we show significant associations between baseline ASI and brain structure a decade later, aligning with often observed cross-sectional associations between PWV and the brain [12, 30, 31, 37, 55]. Therefore, we suggest that ASI is a reasonable surrogate for arterial stiffness as it relates to identification of the overall relationship between arterial stiffness and the brain despite moderate discrepancies in absolute values at the individual-level derived from ASI and PWV observed in a previous study [39]. Future studies may elect to measure arterial stiffness more frequently to uncover details on the temporal relationship between arterial stiffening and subsequent stiffness-related changes in brain structure. Additional information on the time course between arterial stiffness changes and how this may impact the brain could inform potential interventional windows to attenuate vascular related changes in brain structure and function.

In summary, our results point to arterial stiffness at the entry point into older adulthood as more impactful on brain structure 10-years later compared to age-related changes in arterial stiffness. Based on our findings, we propose monitoring and potential interventional approaches to reduce arterial stiffness should occur in midlife to support a healthy trajectory of brain aging into older adulthood.

## Supplementary Materials

The Supplementary data can be found online at: www.aginganddisease.org/EN/10.14336/AD.2023.0419.


