# Supplementary material for: Association of Arterial Stiffness Index and Brain Structure in the UK Biobank: A 10-Year Retrospective Analysis
Source: Aging Dis. 2024 Aug 1;15(4):1872–84. doi: 10.14336/AD.2023.0419 (PMC11272205; doi:10.14336/AD.2023.0419)
Supplement: Supplementary file 1 [file AD-15-4-1872-s.pdf]

SUPPLEMENTARY DATA

Association of Arterial Stiffness Index and Brain Structure in the UK Biobank: A 10-Year Retrospective Analysis

Elric Y. Allison, Baraa K. Al-Khazraji

© 2023. Yang J et al. Published online at http://www.aginganddisease.org/EN/10.14336/AD.2023.0419

SUPPLEMENTARY DATA

Supplemental Table 1. Robust GLM associations between baseline arterial stiffness index and GMV in 50 cortical and 10 subcortical brain regions in a sample of otherwise healthy middle- and older-aged adults, corrected for sex, age, and years between visits (N = 650). *Significant associations are highlighted in bold.*

**N=650**	**Baseline ASI**		**Age**		**Sex**		**Years between Visits**	
**Outcome**	*p*	Holm's corrected *p*	*p*	Holm's corrected *p*	*p*	Holm's corrected *p*	*p*	Holm's corrected *p*
**L. Frontal Pole Volume (mL)**	**0.032**	1.94	**<0.001**	**<0.001**	**<0.001**	**<0.001**	**<0.001**	**0.040**
**R. Frontal Pole Volume (mL)**	0.12	-	**<0.001**	**<0.001**	**<0.001**	**<0.001**	**0.0016**	0.086
**L. Frontal Medial Cortex (mL)**	0.35	-	0.97	-	**0.036**	2.21	0.48	-
**R. Frontal Medial Cortex (mL)**	0.56	-	0.94	-	**0.048**	2.90	0.67	-
**L. Insular Cortex (mL)**	0.67	-	**<0.001**	**<0.001**	**<0.001**	**0.0088**	0.37	-
**R. Insular Cortex (mL)**	0.98	-	**<0.001**	**0.0029**	**<0.001**	**0.0073**	0.28	-
**L. Superior Frontal Gyrus (mL)**	0.20	-	**<0.001**	**<0.001**	**<0.001**	**<0.001**	**0.019**	0.95
**R. Superior Frontal Gyrus (mL)**	**0.042**	2.57	**<0.001**	**<0.001**	**<0.001**	**<0.001**	0.31	-
**L. Middle Frontal Gyrus (mL)**	0.16	-	**<0.001**	**<0.001**	**<0.001**	**<0.001**	**0.020**	0.97
**R. Middle Frontal Gyrus (mL)**	**0.039**	2.41	**<0.001**	**<0.001**	**<0.001**	**<0.001**	**0.0179**	0.91
**L. Inferior Frontal Gyrus Pars Temporalis (mL)**	0.57	-	**<0.001**	**0.013**	**0.03**	1.83	0.51	-
**R. Inferior Frontal Gyrus Pars Temporalis (mL)**	0.083	-	**<0.001**	**<0.001**	0.32	-	0.24	-
**L. Inferior Frontal Gyrus Pars Orbitalis (mL)**	0.81	-	**0.0039**	0.24	0.14	-	0.62	-
**R. Inferior Frontal Gyrus Pars Orbitalis (mL)**	0.20	-	0.44	-	**0.039**	2.38	0.47	-
**L. Anterior Cingulate Gyrus (mL)**	0.48	-	0.16	-	**<0.001**	**0.023**	**0.031**	1.46
**R. Anterior Cingulate Gyrus (mL)**	0.46	-	0.96	-	**<0.001**	**<0.001**	0.44	-
**L. Posterior Cingulate Gyrus (mL)**	0.50	-	**<0.001**	**0.018**	**<0.001**	**<0.001**	0.91	-
**R. Posterior Cingulate Gyrus (mL)**	0.46	-	**0.0026**	0.16	**<0.001**	**<0.001**	0.92	-
**L. Paracingulate Gyrus (mL)**	0.40	-	**<0.001**	**<0.001**	**<0.001**	**0.013**	**0.030**	1.42
**R. Paracingulate Gyrus (mL)**	0.26	-	**<0.001**	**<0.001**	**<0.001**	**0.026**	**0.0051**	0.086
**L. Anterior Superior Temporal Gyrus (mL)**	0.51	-	0.72	-	0.76	-	0.76	-
**R. Anterior Superior Temporal Gyrus (mL)**	0.67	-	0.63	-	0.20	-	0.58	-
**L. Posterior Superior Temporal Gyrus (mL)**	0.18	-	0.96	-	0.87	-	0.12	-
**R. Posterior Superior Temporal Gyrus (mL)**	**<0.001**	**<0.001**	**0.016**	0.96	**0.028**	1.68	0.052	-
**L. Anterior Middle Temporal Gyrus (mL)**	0.63	-	0.38	-	0.77	-	**0.0010**	0.057
**R. Anterior Middle Temporal Gyrus (mL)**	0.96	-	**0.027**	1.63	0.38	-	0.10	-
**L. Posterior Middle Temporal Gyrus (mL)**	0.90	-	**<0.001**	**<0.001**	**<0.001**	**<0.001**	0.47	-
**R. Posterior Middle Temporal Gyrus (mL)**	0.36	-	**<0.001**	**<0.001**	**0.014**	0.87	0.61	-
**L. Anterior Inferior Temporal Gyrus (mL)**	0.27	-	0.57	-	0.43	-	0.17	-

© 2023. Yang J et al. Published online at http://www.aginganddisease.org/EN/10.14336/AD.2023.0419

SUPPLEMENTARY DATA

**R. Anterior Inferior Temporal Gyrus (mL)**	0.18	-	0.67	-	0.098	-	0.86	-
**L. Posterior Inferior Temporal Gyrus (mL)**	0.71	-	**0.0022**	0.14	**<0.001**	**0.0012**	0.56	-
**R. Posterior Inferior Temporal Gyrus (mL)**	0.89	-	**<0.001**	**0.020**	**<0.001**	**<0.001**	0.45	-
**L. Superior Parietal Lobe (mL)**	0.53	-	**<0.001**	**<0.001**	**0.0011**	0.068	0.99	-
**R. Superior Parietal Lobe (mL)**	0.55	-	**<0.001**	**0.0014**	**0.0016**	0.096	0.99	-
**L. Precuneus Cortex (mL)**	0.23	-	**<0.001**	**<0.001**	**<0.001**	**<0.001**	0.20	-
**R. Precuneus Cortex (mL)**	0.20	-	**<0.001**	**<0.001**	**<0.001**	**<0.001**	0.20	-
**L. Anterior Supramarginal Gyrus (mL)**	0.68	-	**0.0022**	0.13	0.14	-	0.44	-
**R. Anterior Supramarginal Gyrus (mL)**	0.30	-	**0.044**	2.71	0.41	-	0.51	-
**L. Posterior Supramarginal Gyrus (mL)**	0.34	-	**<0.001**	**0.019**	**0.0014**	0.084	0.46	-
**R. Posterior Supramarginal Gyrus (mL)**	0.38	-	**<0.001**	**<0.001**	**<0.001**	**<0.001**	0.36	-
**L. Superior Lateral Occipital Lobe (mL)**	**<0.001**	**<0.001**	**<0.001**	**<0.001**	**<0.001**	**<0.001**	**0.0041**	0.22
**R. Superior Lateral Occipital Lobe (mL)**	0.12	-	**<0.001**	**<0.001**	**<0.001**	**<0.001**	0.25	-
**L. Inferior Lateral Occipital Lobe (mL)**	0.60	-	**<0.001**	**<0.001**	**<0.001**	**<0.001**	0.071	-
**R. Inferior Lateral Occipital Lobe (mL)**	0.39	-	**<0.001**	**<0.001**	**<0.001**	**<0.001**	0.31	-
**L. Anterior Parahippocampal Gyrus (mL)**	**0.012**	0.74	**0.032**	1.95	**<0.001**	**0.020**	**<0.001**	**<0.001**
**R. Anterior Parahippocampal Gyrus (mL)**	0.14	-	**0.023**	1.39	0.068	-	**0.0083**	0.43
**L. Posterior Parahippocampal Gyrus (mL)**	0.28	-	0.82	-	0.25	-	0.05	-
**R. Posterior Parahippocampal Gyrus (mL)**	0.59	-	0.57	-	**0.01**	0.59	0.80	-
**L. Thalamus (mL)**	0.53	-	0.16	-	0.40	-	0.41	-
**R. Thalamus (mL)**	0.40	-	0.22	-	**<0.001**	**<0.001**	0.058	-
**L. Hippocampus (mL)**	0.27	-	**0.002**	0.12	**<0.001**	**<0.001**	0.83	-
**R. Hippocampus (mL)**	**0.026**	1.56	**<0.001**	**<0.001**	**<0.001**	0.0072	0.61	-
**L. Amygdala (mL)**	0.33	-	0.20	-	0.078	-	**<0.001**	**<0.001**
**R. Amygdala (mL)**	0.30	-	**0.0040**	0.23	0.87	-	0.69	-

© 2023. Yang J et al. Published online at http://www.aginganddisease.org/EN/10.14336/AD.2023.0419
